# Compost Addition Attenuates the Negative Impacts of High Soil Mineral Nitrogen Levels on Rhizosphere Microbial Characteristics and Enhances Cucumber Growth in Monoculture Systems

**DOI:** 10.3390/plants11131621

**Published:** 2022-06-21

**Authors:** Yune Cao, Yanming Gao, Yongqiang Tian, Jianshe Li

**Affiliations:** 1 College of Agriculture, Ningxia University, Helanshan Xilu No. 489, Yinchuan 750021, China; caohua3221@163.com (Y.C.); myangao@163.com (Y.G.); 2 College of Horticulture, China Agricultural University, Yuanmingyuan West Road No. 2, Haidian District, Beijing 100193, China

**Keywords:** continuously cropped cucumber, rhizosphere, nitrogen cycling, fungal pathogen, plant biomass

## Abstract

Due to the increase in the human population, it is necessary to seek efficient methods of increasing crop productivity and, simultaneously, sustaining the soil. One way is to grow high demand crops continuously without rotating with other crops. This practice is often accompanied by increased rates of fertilizer application that can affect efficient nitrogen (N) cycling in the plant rhizosphere soil which, in turn, affects both plant growth and environmental pollution. In the present study, twelve various cucumber soils were selected from monoculture systems presenting different cropping years and divided into two groups including soils with relatively high mineral N (HMN) content (N > 100 mg kg^−1^ soil) and those with a lower mineral N (LMN) content (N < 100 mg kg^−1^ soil). All soils were amended with the addition of compost alone or in combination with bacterial inoculation to evaluate their effects on plant growth, microbial numbers, N mineralization, and N cycling genes. In general, the HMN soils increased (*p* < 0.05) net N mineralization (NNM) but did not statistically (*p* > 0.05) affect plant biomass compared to the LMN soils; however, compost addition increased both NNM and plant biomass in the HMN soils. In addition, the HMN soils had higher fungal pathogen numbers (FPNs) but lower total microbial biomass (TMB) and bacterial numbers (BNs) compared to the LMN soils; however, compost addition decreased FPNs but increased TMB and BNs in the HMN soils (all *p* < 0.05). Plant biomass was positively related to TMB, BN and NNM but was negatively related to FPN (all *p* < 0.05). In summary, compost addition reduced the high mineral N levels’ adverse effects on the rhizosphere soil and plant growth.

## 1. Introduction

Monoculture is extremely common in agricultural production systems for economically important crops such as vegetables. In addition, this cropping system is becoming more prevalent due to the increased intensity of human intervention on agricultural production through promoting agricultural mechanization and increasing inputs of external resources such as fertilizers and pesticides [1]. In spite of this, monoculture often results in a decrease in crop yields with the passage of time, because there is an increasing trend of soil-borne pathogen-induced disease occurrence, soil microbial activity disturbance, and mineral element imbalance [1,2]. Although a number of practices have been used to alleviate or even reverse the adverse effects of monoculture on crop production [2], this cropping system remains a challenge.

Excessive input of N fertilizers is a common practice used by crop growers in China in order to meet yield demand in monoculture systems [3,4]. Despite this, the N use efficiency of crops tends to reduce, resulting in potential environmental risks due to the leaching of unabsorbed N in the form of nitrate and the emission of N in the volatilized form [4]. Therefore, excessive input of N fertilizers may easily lead to high mineral N levels in soils and thereby induce environmental risks such as groundwater contamination.

In order to maintain a relatively high level of crop yields while at the same time avoiding environmental risks, it is critical to enhance the N use efficiency of crops as well as to inhibit the occurrence of the soil-borne pathogen-induced diseases in monoculture. An efficient practice is to improve soil quality by application of organic composts (OCs). It has been sufficiently demonstrated that OCs have several beneficial effects on soil quality such as the promotion of the capacity of soils to hold water and nutrients, an increase in soil porosity, enhancement of the activity of soil microbes, and the inhibition of the occurrence of the soil-borne pathogen-induced diseases [5,6]. In spite of this, various OCs often have different abilities to inhibit soil-borne pathogen-induced diseases in plants, because there is a need to regulate OCs’ physical and chemical characteristics before their application in crop production systems [6,7]. Thus, the addition of microbial strains that are beneficial to crop growth to OCs is an efficient disease control method when OCs are added into soils to enhance soil quality [7].

Despite the advantages of OCs mentioned above, their promoting effects may not occur in soils characterized by high mineral N concentrations. Under this circumstance, the nutrients (especially N) released by OCs in the mineral form due to the fact of mineralization may not be necessary for plants to absorb, because soil mineral N is enough for plants. Recently, a growing body of research has demonstrated that N cycling in soils is closely associated with the concentration of mineral N, which can be significantly influenced by either the growth status of crops or the activity of soil microbes [8,9]. In specific, if soil mineral N exhibits a low concentration, the N released by OCs in the mineral form may meet the nutrient demands by both crops and soil microbes, resulting in low levels of N losses due to the fact of leaching and emission [8]. Despite this, high inputs of N fertilizers can easily inhibit soil N cycling and increase the occurrence of N leaching and emission [8,9]. Nitrogen cycling is especially important in the plant root rhizosphere, where the efficiency of N uptake is most directly affected. Thus, it is significant to explore the link among soil mineral N levels and N cycling bacteria and enzymes in the rhizosphere as well as to investigate how this relationship is affected by compost addition.

The monoculture in combination with high inputs of N fertilizers often leads to an incredible increase in soil mineral N concentrations [4,10]. The high levels of mineral N concentrations are hypothesized to inhibit the activities of soil microbes and enzymes that are closely associated with N cycling compared to lower levels of mineral N concentrations in soils. Furthermore, it is also hypothesized that the application of OCs attenuate the negative impacts of high mineral N levels on N cycling bacteria activity as measured by enzyme activity and N cycling gene abundance.

The rhizosphere of cucumber can be efficiently used to investigate soil N cycling and its related soil microbes and enzymes, because cucumber soils generally cover a wide range of mineral N concentration in monoculture systems. This crop shows rapid growth and high yield in soils and is widely grown in most regions of the world. The diversity and abundance of N cycling microorganisms occurring in cucumber rhizosphere are poorly understood. Furthermore, causal relationships between these microorganisms and N cycling rates are difficult to predict in the rhizosphere soil [11,12]. Yet, N cycling in rhizosphere soils due to the fact of microbial activity has great potential to affect cucumber growth and other microbial processes as well as potential pollution impacts.

In the present study, twelve various cucumber soils were selected from monoculture systems presenting different cropping years in northern China. These soils have extremely high and variable mineral N contents. The aims of the present study were to decipher (i) whether soils having high mineral N concentrations show different levels of N cycling microorganisms and enzymes and plant biomass than those with lower mineral N concentrations; (ii) how N cycling in cucumber rhizosphere soils with high mineral N content and plant biomass are probably influenced by application of OCs alone or in combination with bacterial inoculation used as disease biological control agent.

## 2. Materials and Methods

### 2.1. Experimental Site Description and Experiment Design

Twelve cucumber soils were selected from monoculture systems presenting various cropping years (1~22 years) in greenhouse vegetable production bases located in the Beijing area (north latitude: 39″26′–41″03′; east longitude: 115″25′–117″30′) in February 2012. The sampling area has an average annual rainfall of approximately 480 mm and an average air temperature of approximately 14.0 °C. The selected soils had various nutrient levels, pH, and electrical conductivities (ECs) under protected cultivation. A description of each site is presented in Table 1. Methods for soil analysis, including the N parameters, are also provided (see below).

According to the concentrations of the mineral N, the selected soils were divided into two groups: soils with a relatively high mineral N (HMN) content (N > 100 mg kg^−1^ soil; range 104–684 mg kg^−1^ soil, mean 279 mg kg^−1^ soil); soils with a lower mineral N (LMN) content (N < 100 mg kg^−1^ soil; range 44.8–78.3 mg kg^−1^ soil, mean 62.5 mg kg^−1^ soil) (Table 1). This grouping method provided an efficient approach for examining the impact of mineral N levels on N cycling in soils.

Soil organic matter (OM) was determined by the dichromate oxidation and titration method [13], and total nitrogen (TN) was analyzed using the classical micro-Kjeldahl method [14]. The pH (soil/water: 1:2.5) was analyzed with a pH meter combined with a glass electrode, and the EC (soil/water: 1:5.0) was measured with an EC meter combined with a glass electrode. Ammonium nitrogen (AN) and nitrate nitrogen (NN) were measured using the methods described in [15,16] after extraction with 2 M KCl. Available phosphorus (AP) was analyzed using the method described in [17] after extraction with 0.5 M NaHCO_3_. Available K was measured as described in [18] after extraction with 1 M NH_4_OAc. The properties of straw compost (SC) used in this study are shown in Table 2. The SC was prepared from plant straw mixed with chicken manure.

*Bacillus subtilis* LY-A, a bacterial strain isolated from cucumber soil and which is beneficial to crop growth and plant disease control, was purchased from the China Center of Industrial Culture Collection. It was prepared as described in [19]. It was used as an inoculant in this study to control disease caused by *Fusarium oxysporum*, and the normal concentration of spores in the culture was 10^9^ spores of *Bacillus subtilis* L^−1^.

Cucumber (*Cucumis sativus* L.) plants were grown in all twelve soils, each of which was treated with different amendments to create four treatments including (i) the untreated soil (S), (ii) the compost-amended soil (SC; 6% compost), (iii) the *Bacillus subtilis*-amended soil (SI; 2.5 × 10^4^ spores g^−1^ soil), and (iv) the compost combined with *Bacillus subtili*-amended soil (SCI; 6% compost + 2.5 × 10^4^ spores g^−1^ soil). For each treatment there were four replicates (four pots per replicate).

Cucumber (*Cucumis sativus* cv. Zhongnong No. 16) seeds were sterilized (soaking for 1.5 min in 3% sodium hypochlorite and for 1.5 min in 70% ethanol, followed by washing with sterile water three times) and then germinated in soils filled in PVC pots (5 kg of soil per pot). The diameter and height of the pot were 25 and 30 cm, respectively. Each pot contained one plant. All pots were placed in a plastic greenhouse having a day/night (26 °C/15 °C) cycle of 10/14 h and an 80% relative humidity.

On day 80 after treatments, plants were harvested, and the rhizosphere soils were sampled by vigorously shaking the root systems of the cucumber plants in order to discard soils that were loosely adhered onto the root surface. After vigorous shaking, each root system held 100–200 g of fresh weight soil (i.e., tightly adhering soil), which was removed from roots, collected, and designated as rhizosphere soil. The rhizosphere soil samples were stored at −20 °C until further analysis. The duration of the experiment was one cropping season in order to compare the response of rhizosphere microbial characteristics and cucumber growth to soil mineral N levels and amendments under the same environment conditions.

### 2.2. DNA Extraction and Quantitative PCR

To extract the genomic DNA of the rhizosphere soil, 0.25 g of fresh sample was treated by the recommended method of the Power Soil DNA Kit produced by MoBio Laboratories Inc. in Sancramento, California, USA. The extracted DNA was loaded on an agarose gel (1%) to check its integrity using electrophoresis. Moreover, its purity was also checked and quantified by a spectrophotometer (NanoDrop ND-1100, Thermo Scientific, Shanghai, China).

The gene abundance was measured using qPCR [20]. The numbers of bacteria, fungi, archaea, *Bacillus subtilis*, and *Fusarium oxysporum* f. sp. *cucumerinum* were measured based on quantifying small subunit rRNA genes. Functional genes measured included the AOA and AOB *amoA* genes for bacterial and archaeal ammonia oxidizers, the *nirK* and *nirS* genes for nitrate reducers, and the *nosZ* gene for denitrifer [12]. *F. oxysporum* f. sp. *cucumerinum* is a major soil-borne pathogen that induces wilt disease in cucumber roots around the world. For all tested genes, the specific primers, fragment lengths, and related thermal profiles are shown in Table 3. The gene abundance was measured through estimating the copy number of a gene in comparison with its standard curve [20].

### 2.3. Nitrogen Mineralization

Nitrogen (N) mineralization of the rhizosphere soil was measured using the incubation method as described in [21]. Briefly, for each replicate approximately 40 g of fresh sample was treated with sterilized deionized water to make its soil water content into 24% (*w*/*w*, dry weight) and then placed in a 4 °C refrigerator for 2 days to obtain equilibrated soils.

The equilibrated soils were further used to analyze net N mineralization. To meet this need, for each replicate, 10 g of soil sample was filled in a 50 mL small flask sealed with a polyethylene film that allows gas but not water to permeate, and then it was placed in a 28 °C incubator for 28 days under dark conditions. Subsequently, for each replicate, both initial (unincubated) and incubated soils were extracted with 2 M KCl to measure AN [15] and NN [16] using the colorimetric method. The net ammonification for a soil was expressed as the difference of AN in the incubated soil comparted to its initial soil, while the net nitrification was the difference of NN (incubated soil vs. initial soil). With respect to net N mineralization, it was expressed as the difference of inorganic N (AN plus NN) (incubated soil vs. initial soil).

### 2.4. Statistical Analysis

SPSS17.0 (SPSS Inc., Chicago, IL, USA) was used to conduct statistical analyses. Soil properties and crop parameters were analyzed by two-way analysis of variance (ANOVA). The factors considered in the ANOVA were soil mineral level, soil amendment treatment, and the interactions of soil mineral level × soil amendment treatment. Once significant differences (*p* ≤ 0.05) were generated by the ANOVA, multiple comparisons were performed by Tukey’s honestly significance difference *post hoc* test (HSD). The log-transformation was applied if the data did not show normality or equal variance. A two-tailed Student’s *t*-test with homogeneous variances was conducted to analyze significant differences in the data between LMN and HMN soils, and *p*-values are presented.

All variables were used to conduct Pearson correlation analysis to obtain the correlation coefficient^®^. To determine the main factors in N cycling genes affecting the N mineralization, canonical correspondence analysis (CCA) [22] in R package vegan (v2.0-8) (University of Oulu, Oulu, Finland) [23] was performed using data matrices of log-transformed N cycling gene abundance data and the variables of N mineralization retained. N cycling gene-related properties (the main matrix) and N mineralization data (the second matrix) were also subjected to multivariate analysis by PC-ORD version 5.0 [24] and plotted using non-metric multidimensional scaling (NMS) to examine whether there is a separation between the high mineral soils and the low mineral N soils, or among the soil amendment treatments (for more details, see [25]).

## 3. Results

### 3.1. Mineral N content

According to the concentration of mineral N (MN), our 12 continuously cropped cucumber soils were divided into two parts, i.e., the high mineral N (HMN) soils (MN > 100 mg kg^−1^) and the low mineral N (LMN) soils (MN < 100 mg kg^−1^). The HMN soils included soils 3, 4, 6, 9, 10, and 11 with an average MN of 279 mg kg^−1^ soil, while the LMN soils included soils 1, 2, 5, 7, 8, and 12 with an average MN of 62.5 mg kg^−1^ soil. Means between LMN and HMN soils were statistically (*p* < 0.05) different for NO_3_^−^-N and total mineral N but not for other variables measured (all *p* > 0.05; Table 1).

### 3.2. Microbial Numbers

The MN levels in the soils significantly influenced the total microbial biomass (TMB) and the numbers of bacteria and *Fusarium oxysporum* (all *p* < 0.05) but not other tested microbial numbers (all *p* > 0.05; Table 4). The TMB and bacterial numbers were statistically lower in the HMN soils compared to the LMN soils (both *p* < 0.05). However, a reverse trend was observed for the number of *Fusarium oxysporum* between these two groups (*p* < 0.05).

Overall, the effects of compost addition on the tested microbial numbers (SC vs. S, SCI vs. SI) was more pronounced than that of bacteria inoculation (compare SI vs. S and SCI vs. SC) (Table 4). The total microbial biomass, bacterial numbers, and fungal numbers were statistically increased by compost addition (all *p* < 0.05; SC vs. S; SCI vs. SI). The abundance of *Fusarium oxysporum* f. sp. *cucumerinum* statistically (*p* < 0.05) decreased by all soil amendment treatments in the HMN soils (SC, SCI, and SI vs. S).

### 3.3. Nitrogen Cycling Genes

To examine whether the HMN soils had different abundances of N cycling microbes and enzymes in cucumber rhizosphere than the LMN soils, we analyzed the possible differences in N cycling genes in response to soil mineral N levels (Table 5). The LMN soils did not show a statistically significant (*p* = 0.475) difference in AOB *amoA* gene abundance but higher (*p* < 0.001) AOA *amoA* abundance than the HMN soils, resulting in a relatively low AOB/AOA ratio in the LMN soils. Furthermore, the LMN soils showed a significantly (*p* = 0.016) higher *nirS* abundance but did not show a statistically significant difference in the abundances of *nirK* and *nosZ* genes (both *p* > 0.05) compared to HMN soils. In contrast, the compost addition generally increased (*p* < 0.05) the *nirK* and *nosZ* gene abundances but had no impact (*p* > 0.05) om the *nirS* abundance (compare SC vs. S and SCI vs. SI). Although the compost addition had no effect (*p* > 0.05) on the *amoA* gene abundance of either AOB or AOA, the AOB: AOA ratio was statistically (*p* < 0.05) lower in the compost-treated HMN soils compared to the untreated HMN soils. In general, there was no effect of bacterial inoculation on the N cycling-related gene abundances and the AOB: AOA ratio (SI vs. S and SCI vs. SC).

### 3.4. Nitrogen Mineralization

**Figure 1:**
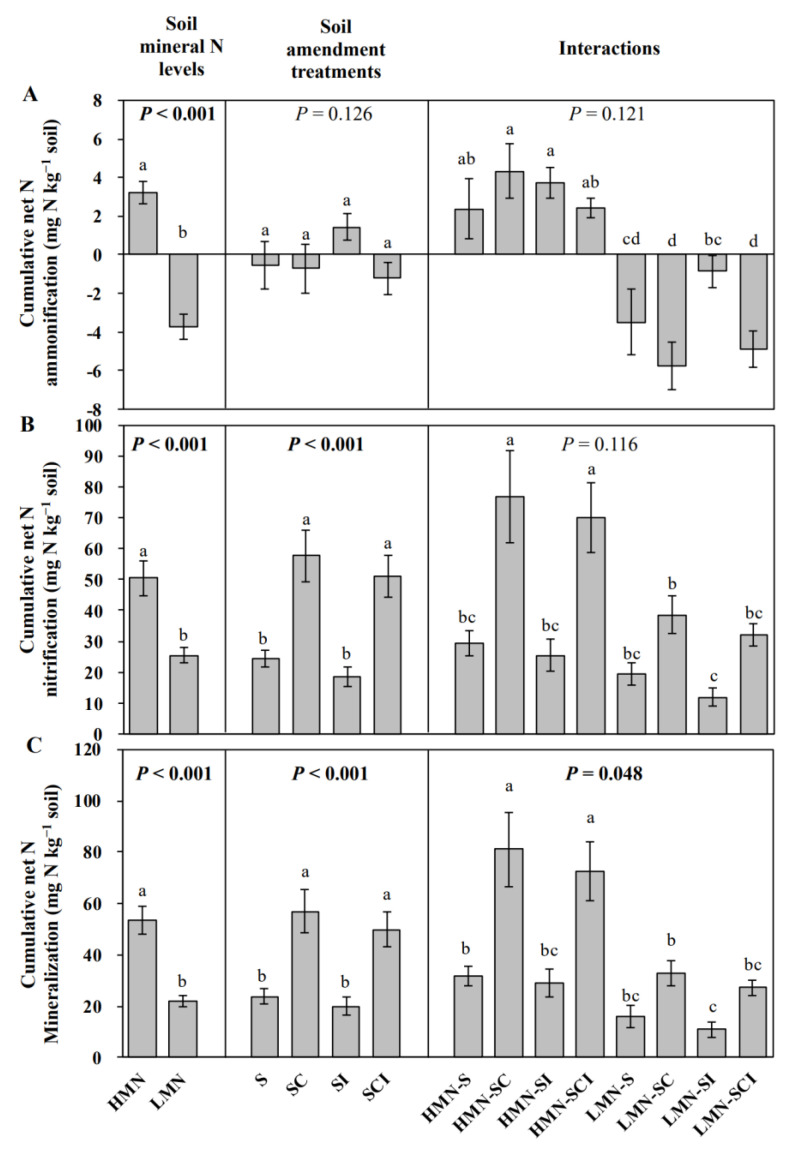
Net N ammonification (**A**), nitrification (**B**), and mineralization (**C**) in the high (HMN) and low mineral N (LMN) soils under the S (control soil), SC (soil treated with straw compost), SI (soil treated with bacterial inoculation), and SCI (soil treated with straw compost combined with bacterial inoculation) treatments. Different letters denote a significant difference (*p* ≤ 0.05). *p*-Values in bold text denote significant differences among treatments (*p* < 0.05). Bars denote the means ± SE.

In general, the soil mineral N level significantly affected all these N cycling processes (all *p* < 0.001), and the HMN soils showed significantly higher cumulative N ammonification (Figure 1A) and nitrification (Figure 1B) than the LMN soils, resulting in relatively high cumulative N mineralization in the HMN soils (Figure 1C). Interestingly, the cumulative net N ammonification value was positive in the HMN soils but was negative in the LMN soils (Figure 1A). The cumulative net N nitrification and mineralization were higher in the compost-treated soils as compared to untreated soils, but this trend was only significant (*p* < 0.05) in the HMN soils (SC vs. S and SCI vs. SI; Figure 1B,C). In general, no effect of bacterial inoculation on the tested N cycling processes was found (SI vs. S and CI vs. SC).

### 3.5. Relationships between N Cycling Genes and N Mineralization

The HMN soils generally had different levels of N cycling genes and processes than the LMN soils in cucumber rhizosphere. Thus, we further examined whether the relationship between N mineralization and the abundance of genes associated with N cycling in the HMN and LMN soils was different. Net N ammonification was significantly correlated with the abundance of *amoA* gene of either AOA or AOB (both *p* < 0.001) but not the *nirK* and *nirS* gene abundances (both *p* > 0.05) in the HMN soils. In contrast, a reverse trend was observed in the LMN soils (Table 6). Furthermore, net N ammonification showed a significant and negative correlation with the *nosZ* abundance in the HMN soils (*r* = −0.405, *p* < 0.001) but a significant and positive correlation with the *nosZ* abundance in the LMN soils (*r* = 0.384, *p* < 0.001) (Table 6). It was noted that, although both *nirK* and *nirS* are genes for nitrate reducers, reverse relationships were found between these two genes and the net N ammonification in the LMN soils. In general, no statistical (*p* > 0.05) correlation was found among the abundances of tested N cycling genes and net N nitrification and mineralization in both HMN and LMN soils (Table 6). Since each N cycling process is generally affected by more than one gene, the net N mineralization is generally affected by the equilibrium of different related genes rather than one specific gene. Thus, we further calculated the ratios of different related genes and found that net N nitrification and mineralization were significantly positively correlated with the *nosZ*/*nirS* and *nirK*/*nirS* ratios in the HMN soils (all *p* < 0.001) but significantly negatively correlated with the AOB/AOA ratio in the LMN soils (*p* < 0.05) (Table 6).

**Figure 2:**
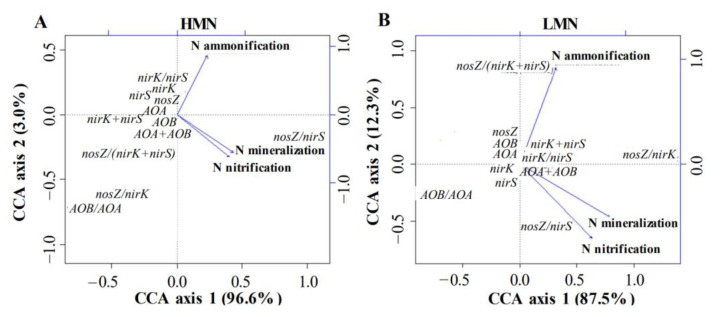
CCA plots for N cycling genes and N mineralization associated with cucumber rhizosphere soils with (**A**) high (HMN) or (**B**) low mineral N (LMN) levels. The diagrams representing the CCA models were generated with standardized parameters based on F-statistic significant tests. The blue lines’ length and angle were used to reflect correlations among CCA axes and N mineralization processes. *amoA* denotes the gene for ammonia oxidizing (AO) bacteria (AOA) or archaea (AOB); *nirK* and *nirS* represent genes for nitrate reducers; *nosZ* is the gene for denitrifer.

To further confirm the result that the N mineralization was affected by the equilibrium of different related genes rather than one specific gene, we performed canonical correspondence analysis (CCA) to determine the main factors in N cycling genes affecting the N mineralization (Figure 2). The results of CCA showed that in both the HMN and LMN soils, the net N ammonification, nitrification, and mineralization were mainly affected by the ratios of different related genes rather than one specific gene. N ammonification was mainly affected by the AOB/AOA and *nosZ*/*nirK* ratios in the HMN soils (Figure 2A), but mainly affected by the *nosZ*/(*nirK* + *nirS*) ratio in the LMN soils (Figure 2B). In both the HMN and LMN soils, N nitrification and mineralization were mainly affected by the *nosZ*/*nirS* ratio.

### 3.6. Effects of Soil Mineral N Levels and Soil Amendments on N Cycling

**Figure 3:**
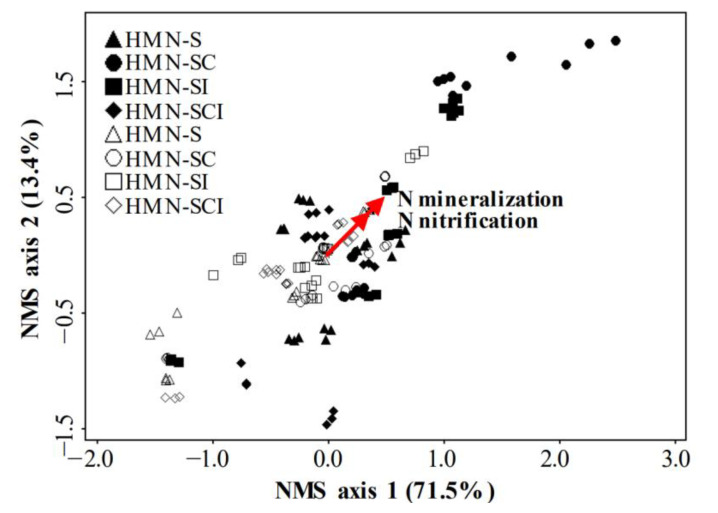
NMS plots for N cycling gene-related properties (the main matrix) and N mineralization data (the second matrix) (*n* = 144) in the high (HMN) and low mineral N (LMN) soils under the S (control soil), SC (soil treated with straw compost), SI (soil treated with bacterial inoculation), and SCI (soil treated with straw compost combined with bacterial inoculation) treatments. The red lines’ length and angle were used to reflect correlations between NMS axes and the N nitrification and mineralization.

To examine whether there is a separation between the HMN soils and the LMN soils, or among soil amendment treatments in N cycling in cucumber rhizosphere soils, we subjected N cycling gene-related properties and N mineralization data to multivariate analysis and plotted using nonmetric multidimensional scaling (NMS) (Figure 3). The results of NMS showed that the N nitrification and mineralization were the main factors influencing the final ordination solution. Generally, under the same treatment, there was a clear separation between the HMN (closed symbols) and LMN soils (open symbols). Compost addition induced the greatest changes in N cycling in cucumber rhizosphere soils (SCI vs. SI and SC vs. S). However, the effect of bacterial inoculation on changes in soil N cycling was generally not obvious.

### 3.7. Plant Biomass

**Figure 4:**
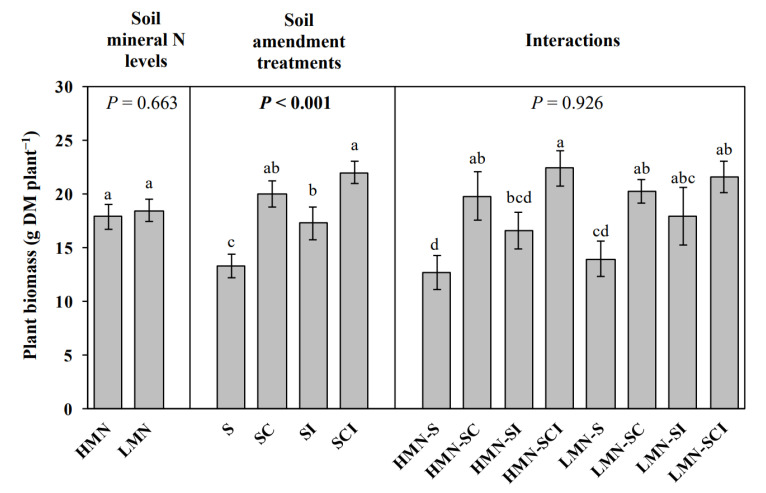
Plant biomass in the high (HMN) and low mineral N (LMN) soils under the S (control soil), SC (soil treated with straw compost), SI (soil treated with bacterial inoculation), and SCI (soil treated with straw compost combined with bacterial inoculation) treatments. Different letters denote a significant difference (*p* ≤ 0.05). *p*-Values in bold text denote the significant differences among treatments (*p* < 0.05). Bars denote the means ± SE. DM represents dry matter.

Plant biomass was significantly affected with the soil amendment treatments (*p* < 0.001) rather than the mineral N level of soils (*p* = 0.663) (Figure 4).

On average, plants were larger (e.g., greater plant biomass) in the compost-treated soils compared to untreated soils (SC vs. S and SCI vs. SI). Similarly, the plant biomass was generally increased by bacteria inoculation (SI vs. S and SCI vs. SC). Plant biomass had a positive correlation with the total microbial biomass, bacteria, and net N mineralization but negatively correlated with archaea and *Fusarium oxysporum* f. sp. *cucumerinum* (all *p* < 0.05; Table 7).

## 4. Discussion

We investigated rhizosphere soil N cycling of monoculture cucumbers growing in soils that represented various cropping years. For cucumber rhizosphere soil, N cycling was mainly affected by soil mineral N levels. Thus, we quantified how soil mineral N levels influenced microbial populations, N cycling genes, and net N mineralization occurring in cucumber rhizosphere soils.

### 4.1. Soil Mineral N Level and Microbial Population

Interestingly, the total microbial biomass and bacterial numbers showed an increasing trend in HMN soils compared to LMN soils, but a reverse trend was found for the number of *Fusarium oxysporum* (Table 4). Similarly, several previous studies also showed that a higher fertility level might induce more disease in plants [26,27]. Although the related mechanisms have remained undeciphered, it is indeed that plant diseases can be affected by the nitrogen form [26]. Additionally, greater *Fusarium oxysporum* numbers in HMN soils probably resulted from the reduction of either total microbial biomass or bacterial numbers, which may lead to decreased microbial diversity [28]. A recent study [29] demonstrated the critical role played by soil microbial diversity in determining the occurrence of microbial pathogens in soils.

### 4.2. Soil Mineral N Level and N Cycling

The HMN soils had different abundances of N cycling genes than the LMN soils. Generally, the HMN soils had lower abundances of the AOA *amoA* and *nirS* genes (Table 5) but higher net N ammonification, nitrification, and mineralization (Figure 1) compared with the LMN soils. This is inconsistent with established biochemical and microbial physiology principles, because if there is abundant mineral N present, there is no need for microorganisms to expend energy to mineralize and nitrify organic N [30]. In fact, in these HMN soils, the level of mineral N was extremely high compared to many soils where N cycling studies have been conducted [12,31] and, thus, the need for N mineralization in our soils would be even less required.

Surprisingly, although both the AOA and AOB mediate the same microbial process (i.e., ammonia-oxidation), the AOB *amoA* abundance was not statistically (*p* = 0.475) affected by the soil mineral N level (Table 5). Since ammonia oxidation was the first step limiting the nitrification rate, it was long thought that this process was driven by the bacterial *amoA* gene alone [32]. However, there is increasing evidence that archaeal ammonia monooxygenase genes are distributed widely in marine and terrestrial environments [33,34,35]. This has resulted in a revision in the current knowledge regarding N cycling in soils.

The abundance of AOA often exceeds that of AOB in soil systems [34]. However, the present study showed that the *amoA* had a greater abundance in AOB compared to AOA in both the HMN and LMN soils (Table 5). The results of several recent advanced studies have shown that AOB was often predominant in agricultural soils with a high fertility level [31,36]. Furthermore, the AOA community often has a greater abundance in soils with low fertility level [37,38], similar to our finding that the LMN soils had a greater abundance of AOA *amoA* gene compared to the HMN soils (Table 5). This may have been due to the fact that the AOA community tends to thrive in environments that are more nutrient limiting [39]

The *nirS* and *nirK* genes encode nitrite reductases with similar functions. In this study, the soil mineral N levels significantly (*p* = 0.016) affected *nirS* abundance but not *nirK* abundance (*p* = 0.980) in the cucumber rhizosphere (Table 5). Several recent studies have suggested that the microorganisms carrying *nirS* and *nirK* have different patterns of resource utilization or different habitat preferences [12,40,41]. Our results suggest that the denitrifiers carrying *nirS* are more adapted to low mineral N conditions, while the *nirK* bearing denitrifiers are relatively stable in cucumber rhizosphere soils with different mineral N levels. In addition, the HMN and LMN soils in this study contrasted with respect to soil mineral N level (*p* < 0.05) but not the other tested soil properties (e.g., pH, EC, and most tested nutrients; all *p* > 0.05; Table 1), indicating that soil mineral N levels may have a profound influence on the composition of the denitrifying communities (e.g., the average *nirK*/*nirS* ratio in the HMN and LMN soils was 6.28 and 2.84, respectively; *p* < 0.001). Several studies have verified that *nirS* rather than *nirK* plays an important role in soils under wet conditions [12,42,43]. Similar to this, our results suggest that *nirS* rather than *nirK* plays a critical role in soils characterized by low mineral N content (Table 5).

Recent findings have shown that the abundance of genes associated with N cycling can be used to predict the rates of potential nitrification and denitrification [12]. Since the net N nitrification had a significant correlation with the *nosZ*/*nirS* and *nirK*/*nirS* ratios in the HMN soils (both *p* < 0.001; Table 6), the increased net N nitrification in cucumber rhizosphere soil may be mediated by the equilibrium between *nosZ* and *nirS* and/or between *nirK* and *nirS* under soil environments with high mineral N.

### 4.3. Soil Amendment and N Cycling

Compost addition affected the N cycling in cucumber rhizosphere soils. Generally, the compost-treated soils showed not only higher abundances of the *nirK* and *nosZ* genes (Table 5) but also higher net N nitrification and mineralization (Figure 1) (SCI vs. SI and SC vs. S). Because the addition of compost resulted in a significant (*p* < 0.001) increase in *nirK* abundance but not *nirS* abundance (Table 5), *nirK* but not *nirS* plays an important ecological role in soils treated with compost. The *NosZ* gene has been demonstrated as a strong predictor for potential denitrification rates, because its abundance was significantly correlated to potential denitrification rates [12]. This gene can also be strongly affected by environmental factors such as soil organic matter and nitrate [12,40,42]. In this study, the *nosZ* gene was only significantly affected by the compost addition in the LMN soils but not in the HMN soils (Table 5), which means that the compost addition combined with a low soil mineral N level may enhance potential denitrification rates.

### 4.4. The Responses of Plant Biomass to Mineral N Level, Amendments and N Cycling

Although the soil mineral N level significantly affected the net N ammonification, nitrification, and mineralization (all *p* < 0.001; Figure 1), it did not statistically affect the plant biomass at harvest (*p* = 0.663; Figure 4). This means that, when mineral N content is already high in soils, production of mineral N via N mineralization may exceed the N uptake demands of plants and microorganisms in plant rhizosphere [8], ultimately resulting in increases in soil ecosystem N loss [8,9]. As previously stated, this seems contrary to what would be expected, since the microbial population in rhizospheres of the HMN soils would not need to continue to mineralize N if sufficient amounts were already present. On the other hand, even if plant growth may benefit from soils having high levels of mineral N, the potential of high mineral N level to cause more plant disease may result in negative effect on plant growth (Table 4).

The trend in continued mineralization of N, even in the HMN soils, can be reversed, however, if the HMN soils are amended with compost. Indeed, the compost addition significantly increased both the plant biomass (*p* < 0.05; Figure 4) and net N nitrification in the HMN soils (*p* < 0.05; Figure 1). The increased net N nitrification in cucumber rhizosphere soils may result from the increased N uptake demands induced by plant growth [8], removing the nitrate-N pool from the rhizosphere under compost addition treatments compared to the untreated control. The result would be microorganisms striving to restore nitrate in the rhizosphere and, thus, a stimulation of nitrification. In addition, although a high mineral N level has the potential to cause more plant disease, this could be reduced by compost addition (Table 4).

## 5. Conclusions

In summary, soil mineral N levels strongly influenced N cycling in cucumber rhizosphere soils. Although the high mineral N level tended to increase the net N mineralization, it had the potential to cause more plant disease but less microbial biomass and bacterial numbers in cucumber rhizosphere soil. Plant biomass was not closely associated with the mineral N level of soils, but it could be increased by amending soils with compost addition, which increased net N mineralization but decreased fungal pathogen numbers in cucumber rhizosphere soil.

## Figures and Tables

**Table 1:** The selected properties of twelve cucumber soils from various cropping years.

Mineral N Level	Soil Codes ^a^	Cropping Years	Organic Matter	Total Nitrogen	Ammonium Nitrogen	Nitrate Nitrogen	Mineral Nitrogen	Available Phosphorus	Available Potassium	pH	Electrical Conductivity
			(g kg^−1^)	(g kg^−1^)	(mg kg^−1^)	(mg kg^−1^)	(mg kg^−1^)	(mg kg^−1^)	(mg kg^−1^)		(mS cm^−1^)
LMN ^a^	Soil-1	20 years	25.6 ± 0.8	1.57 ± 0.09	6.63 ± 0.28	50.8 ± 0.8	57.4 ± 3.4	144 ± 5	599 ± 27	7.09 ± 0.48	0.50 ± 0.01
	Soil-2	16 years	9.10 ± 0.1	0.55 ± 0.02	12.4 ± 0.42	60.5 ± 3.6	72.9 ± 4.6	148 ± 5	450 ± 19	7.16 ± 0.07	0.39 ± 0.03
	Soil-5	22 years	29.3 ± 1.1	2.66 ± 0.17	11.2 ± 0.17	33.6 ± 1.3	44.8 ± 2.4	104 ± 6	314 ± 9	7.52 ± 0.37	0.50 ± 0.01
	Soil-7	7 years	13.7 ± 1.0	0.84 ± 0.03	7.22 ± 0.04	42.0 ± 1.7	49.2 ± 1.7	79.1 ± 2.9	31.0 ± 2	7.46 ± 0.05	0.40 ± 0.02
	Soil-8	1 year	15.3 ± 1.1	0.93 ± 0.01	7.32 ± 0.40	64.9 ± 4.0	72.2 ± 2.8	167 ± 1	48.3 ± 0.1	7.44 ± 0.35	0.57 ± 0.02
	Soil-12	3 years	19.2 ± 1.0	1.18 ± 0.03	8.02 ± 0.35	70.3 ± 4.5	78.3 ± 0.3	176 ± 11	132 ± 3	6.75 ± 0.47	0.84 ± 0.03
HMN ^b^	Soil-3	10 years	19.0 ± 0.3	1.16 ± 0.02	16.8 ± 0.4	285 ± 6	303 ± 16	113 ± 3	500 ± 6	7.15 ± 0.23	1.30 ± 0.09
	Soil-4	4 years	25.6 ± 0.9	1.89 ± 0.03	8.40 ± 0.38	96.1 ± 1.1	104 ± 7	66.1 ± 4	374 ± 21	7.31 ± 0.35	0.61 ± 0.02
	Soil-6	15 years	11.8 ± 0.1	0.76 ± 0.03	10.4 ± 0.5	143 ± 11	153 ± 9	131 ± 2	238 ± 12	7.51 ± 0.14	0.76 ± 0.01
	Soil-9	18 years	20.8 ± 1.2	1.27 ± 0.02	12.8 ± 0.2	241 ± 14	254 ± 16	341 ± 7	293 ± 20	6.28 ± 0.07	1.75 ± 0.03
	Soil-10	12 years	27.8 ± 0.8	2.90 ± 0.17	27.1 ± 0.8	657 ± 21	684 ± 27	358 ± 5	687 ± 30	5.87 ± 0.15	5.52 ± 0.43
	Soil-11	5 years	20.3 ± 0.2	1.21 ± 0.57	14.9 ± 0.6	162 ± 6	177 ± 14	137 ± 2	230 ± 2	6.41 ± 0.43	1.17 ± 0.07
Means ^c^	LMN		18.7 ± 3.1	1.29 ± 0.31	8.80 ± 0.98	53.7 ± 5.8	62.5 ± 5.7	136 ± 15	262 ± 94	7.24 ± 0.12	0.53 ± 0.07
	HMN		20.9 ± 2.3	1.53 ± 0.30	15.1 ± 2.7	264 ± 83	279 ± 86	191 ± 51	387 ± 73	6.76 ± 0.27	1.85 ± 0.75
*p*-Value			0.583	0.591	0.054	**0.031**	**0.031**	0.330	0.320	0.133	0.111

^a^ Soils 1, 2, 5, 7, 8, and 12 were designated low mineral N (LMN) soils (total mineral N < 100 mg kg^−1^ soil). ^b^ Soils 3, 4, 5, 9, 10, and 11 were designated high mineral N (HMN) soils (total mineral N > 100 mg kg^−1^ soil). ^c^ Means between LMN and HMN soils were significantly (*p* < 0.05) different for NO_3_^−^-N and total mineral N but not for other variables measured.

**Table 2:** The characteristics of the straw compost used to amend soils.

DNA Extract	Microbial Properties ^a^ (Gene Copies (g^−1^ Dry Compost))							
(μg DNA g^−1^ Dry Compost)	Bacteria	Fungi	Archaea	AOB *amoA*	AOA *amoA*	*nirK*	*nirS*	*nosZ*
16.26 ± 3.14	7.05 × 10^12^ ± 0.52 × 10^12^	1.25 × 10^12^ ± 0.02 × 10^12^	2.76 × 10^10^ ± 0.06 × 10^10^	6.20 × 10^8^ ± 0.12 × 10^8^	1.01 × 10^7^ ± 0.07 × 10^7^	2.54 × 10^8^ ± 0.11 × 10^8^	7.75 × 10^9^ ± 0.29 × 10^9^	7.87 × 10^9^ ± 0.09 × 10^9^
Organic carbon	Total nitrogen	Ammonium nitrogen	Nitrate nitrogen	Mineral nitrogen	Available phosphorus	Available potassium	pH	Electrical conductivity
(g kg^−1^)	(g kg^−1^)	(g kg^−1^)	(g kg^−1^)	(g kg^−1^)	(g kg^−1^)	(g kg^−1^)		(mS cm^−1^)
297.9 ± 6.3	17.11 ± 0.46	0.17 ± 0.01	1.90 ± 0.09	2.07 ± 0.14	3.58 ± 0.23	16.35 ± 1.31	6.25 ± 0.21	4.05 ± 0.03

^a^ The abundances of various genes were measured using qPCR. The numbers of bacteria, fungi, and archaea were measured based on quantifying small subunit rRNA genes. *amoA* denotes the gene for ammonia oxidizing (AO) bacteria (AOA) or archaea (AOB); *nirK* and *nirS* represent genes for nitrate reducers; *nosZ* is the gene for denitrifer.

**Table 3:** The specific primers and qPCR conditions for the tested genes.

Target	Primer Name	Primer Sequence 5’ to 3’	Fragment Length	Thermal Profile	Number of Cycles	Reference
Bacteria	Eub338	ACTCCTACGGGAGGCAGCAG	181 bp	95 °C-5 min	1	Fierer et al. (2005) Petersen et al. (2012)
	Eub518	ATTACCGCGGCTGCTGG		95 °C-60 s/53 °C-30 s/ 72 °C-60 s	40	
Fungi	5.8 s	CGCTGCGTTCTTCATCG	300 bp	95 °C-5 min	1	Fierer et al. (2005)
	ITS1f	TCCGTAGGTGAACCTGCGG		95 °C-60 s/53 °C-30 s/ 72 °C-60 s	40	
Archaea	Arch344	TTCGCGCCTGSTGCRCCCCG	572 bp	95 °C-3 min	1	Mori et al. (2003) Petersen et al. (2012)
	Arch915	GTGCTCCCCCGCCAATTCCT		95 °C-30 s/65 °C-30 s/ 72 °C-45 s	40	
AOB amoA	amoA1F	GGGGTTTCTACTGGTGGT	491 bp	94 °C-5 min	1	Rotthauwe et al. (1997) Petersen et al. (2012)
	amoA2R	CCCCTCKGSAAAGCCTTCTTC		94 °C-30 s/55 °C-45 s/ 72 °C-60 s	40	
AOA amoA	Arch-amoAF	STAATGGTCTGGCTTAGACG	635 bp	94 °C-5 min	1	Francis et al. (2005) Petersen et al. (2012)
	Arch-amoAR	GCGGCCATCCATCTGTATGT		94 °C-30 s/53 °C-45 s/ 72 °C-60 s	40	
*nirK*	NirK876 NirK1040	ATYGGCGGVAYGGCGA GCCTCGATCAGRTTRTGGTT	165 bp	95 °C-5 min	1	Henry et al. (2004) Petersen et al. (2012)
				95 °C-15 s/63–58 °C-30 s/ 72 °C-30 s	6 (touchdown)	
				95 °C-15 s/58 °C-30 s/72 °C-30 s	40	
*niS*	Cd3F	GT(C/G)AACGT(C/G)AAGGA(A/G) AC(C/G)GG	407 bp	95 °C-5 min 95 °C-15 s/63–58 °C-30 s/ 72 °C-30 s 95 °C-15 s/58 °C-30 s/72 °C-30 s	1 6 (touchdown) 40	Michotey et al. (2000) Throbäck et al. (2004) Petersen et al. (2012)
	R3cd	GA(C/G)TTCGG(A/G)TG(C/G)GTCT TGA				
*nosZ*	NosZ2F NosZ2R	CGCRACGGCAASAAGGTSMSSGT CAKRTGCAKSGCRTGGCAGAA	267 bp	95 °C-5 min	1	Henry et al. (2006) Petersen et al. (2012)
				95 °C-30 s/65–60 °C-30 s/ 72 °C-30 s	6 (touchdown)	
				95 °C-15 s/60 °C-15 s/ 72 °C-30 s	40	
*Bacillus*	Bsub5F	AAGTCGAGCGGACAGATGG	595 bp	95 °C-5 min	1	Wattiau et al. (2001)
*subtilis*	Bsub3R	CCAGTTTCCAATGACCCTCCCC		95 °C-60 s/65 °C-30 s/ 72 °C-60 s	40	
*F. oxysporum* f. sp. *cucumerinum*	FocF3 FocR7	AAACGAGCCCGCTATTTGAG TATTTCCTCCACATTGCCATG	244 bp	95 °C-5 min 95 °C-15 s/60 °C-45 s/ 72 °C-60 s	1 40	Lievens et al. (2007)

**Table 4:** Total microbial biomass and the number of selected microorganisms as affected by soil mineral N levels and soil amendment treatments.

Treatments ^a^	Total Microbial Biomass ^b^	Selected Microorganisms (Genes g^−1^ Soil)				
	(×10^3^ DNA ng g^−1^ Soil)	Bacteria (×10^9^)	Fungi (×10^9^)	Archaea (×10^8^)	*Bacillus subtilis* (×10^8^)	*Fusarium oxysporum* f. sp. *Cucumerinum* (×10^5^)
Soil mineral N levels:						
HMN	9.57 b ^c^	10.61 b	7.29	6.91	1.61	15.01 a
LMN	11.01 a	14.12 a	7.81	9.53	1.93	1.53 b
*p*-Value ^d^	**0.000**	**0.000**	0.450	0.096	0.091	**0.038**
Soil amendment treatments:						
S	8.16 c	10.23 b	5.15 b	10.01	1.91 ab	26.52 a
SC	12.02 a	14.36 a	10.01 a	6.10	1.43 b	2.03 b
SI	9.18 b	10.21 b	6.09 b	9.41	2.12 a	1.89 b
SCI	11.71 a	14.50 a	8.93 a	7.23	1.51 b	2.59 b
*p*-Value	**0.000**	**0.000**	**0.000**	0.235	**0.019**	**0.024**
Interactions:						
HMN-S	7.77 e	8.40 c	5.85 cd	8.40 ab	1.41 bc	51.90 a
HMN-SC	11.53 b	12.04 bc	9.03 ab	4.30 b	1.00 c	2.57 b
HMN-SI	8.99 cde	9.85 bc	7.65 bc	9.11 ab	2.42 a	2.79 b
HMN-SCI	10.10 c	12.23 b	6.63 bcd	5.82 ab	1.49 bc	2.66 b
LMN-S	8.55 de	12.02 b	4.46 d	11.71 a	2.45 a	1.11 b
LMN-SC	12.60 ab	16.60 a	11.02 a	8.03 ab	1.87 ab	1.49 b
LMN-SI	9.36 cd	10.62 bc	4.54 d	9.70 ab	1.85 ab	0.99 b
LMN-SCI	13.31 a	16.91 a	11.21 a	8.52 ab	1.44 bc	2.53 b
*p*-Value	**0.007**	0.398	**0.001**	0.893	**0.008**	**0.019**

^a^ HMN: high mineral nitrogen; LMN: high mineral nitrogen; S: control soil; SC: soil treated with straw compost; SI: soil treated with bacterial inoculation; SCI: soil treated with straw compost combined with bacterial inoculation. ^b^ Total microbial biomass was represented by extracted DNA. ^c^ Different letters in the same column denote a significant difference (*p* ≤ 0.05). ^d^ *p*-Values in bold text denote significant differences among treatments (*p* < 0.05).

**Table 5:** Abundance of selected nitrogen cycling genes as affected by soil mineral N levels and soil amendment treatments.

Treatments ^a^	Selected N Cycling Genes ^b^ (Genes g^−1^ Soil)					AOB/AOA
	AOB *amoA* (×10^6^)	AOA *amoA* (×10^6^)	*nirK* (×10^7^)	*nirS* (×10^7^)	*nosZ* (×10^7^)	
Soil mineral N levels:						
HMN	4.52	2.24 b ^c^	3.80	2.34 b	6.16	1.92 a
LMN	5.00	3.32 a	3.79	4.67 a	6.81	1.46 b
*p*-Value ^d^	0.475	**0.000**	0.980	**0.016**	0.236	**0.029**
Soil amendment treatments:						
S	4.90	2.61	3.03 b	4.13	5.95 b	1.79 a
SC	4.45	2.92	4.34 a	2.73	7.79 a	1.45 b
SI	5.77	2.68	3.51 b	3.78	5.56 b	2.13 a
SCI	3.92	2.90	4.29 a	3.40	6.63 ab	1.39 b
*p*-Value	0.256	0.653	**0.000**	0.757	**0.023**	**0.048**
Interactions:						
HMN-S	4.50 ab	2.02 d	3.42 c	1.51 bc	6.14 abc	2.01 a
HMN-SC	3.70 b	2.38 cd	4.50 ab	0.88 c	7.32 abc	1.47 b
HMN-SI	6.55 a	2.10 d	4.05 bc	2.26 bc	5.84 bc	2.76 a
HMN-SCI	3.32 b	2.45 bcd	3.22 cd	4.71 ab	5.34 c	1.43 b
LMN-S	5.30 ab	3.20 abc	2.65 d	6.76 a	5.76 c	1.57 b
LMN-SC	5.20 ab	3.46 a	4.19 bc	4.56 abc	8.27 a	1.44 b
LMN-SI	5.00 ab	3.27 ab	2.96 d	5.30 ab	5.29 c	1.49 b
LMN-SCI	4.51 ab	3.35 a	5.36 a	2.08 bc	7.91 ab	1.34 b
*p*-Value	0.369	0.966	**0.000**	**0.025**	0.151	**0.041**

^a^ HMN: high mineral nitrogen; LMN: high mineral nitrogen; S: control soil; SC: soil treated with straw compost; SI: soil treated with bacterial-inoculation; SCI: soil treated with straw compost combined with bacterial inoculation. ^b^ *amoA* denotes the gene for ammonia oxidizing (AO) bacteria (AOA) or archaea (AOB); *nirK* and *nirS* represent genes for nitrate reducers; *nosZ* is the gene for denitrifer. ^c^ Different letters in the same column denote a significant difference (*p* ≤ 0.05). ^d^ *p*-Values in bold text denote the significant differences among treatments (*p* < 0.05).

**Table 6:** Pearson correlations coefficient (*r*) reflecting the responses of N cycling genes to net N ammonification, nitrification, and mineralization (*n* = 24).

N Cycling Genes ^a^	HMN ^b^			LMN		
	Net N Ammonification	Net N Nitrification	Net N Mineralization	Net N Ammonification	Net N Nitrification	Net N Mineralization
AOB *amoA*	−0.395 *** ^c^	−0.137	−0.180	−0.213	−0.144	−0.221
AOA *amoA*	−0.500 ***	−0.005	−0.057	0.119	0.053	0.093
AOB + AOA	−0.446 ***	−0.119	−0.167	−0.130	−0.097	−0.145
*nirK*	−0.057	0.131	0.126	0.276 *	−0.047	0.030
*nirS*	−0.175	−0.019	−0.038	−0.259 *	0.143	0.081
*nirK* + *nirS*	−0.208	0.018	−0.203	−0.222	−0.162	0.113
*nosZ*	−0.405 ***	−0.113	−0.157	0.384 ***	0.006	0.120
AOB/AOA	−0.270 *	−0.151	−0.181	−0.267 *	−0.248 *	−0.351 **
*nosZ*/*nirK*	−0.408 ***	−0.149	−0.194	0.076	0.142	0.179
*nosZ*/*nirS*	0.202	0.479 ***	0.506 ***	−0.089	0.193	0.187
*nosZ*/(*nirK + nirS*)	−0.275 *	−0.079	−0.108	0.375 ***	−0.097	0.004
*nirK*/*nirS*	0.315 **	0.448 ***	0.487 ***	−0.172	0.231	0.203

^a^*amoA* denotes the gene for ammonia oxidizing (AO) bacteria (AOA) or archaea (AOB); *nirK* and *nirS* represent genes for nitrate reducers; *nosZ* is the gene for denitrifer. ^b^ HMN: high mineral nitrogen; LMN: high mineral nitrogen. ^c^ Values in bold are statistically significant at *p* < 0.05. * *p* < 0.05, ** *p* < 0.01, and *** *p* < 0.001.

**Table 7:** Pearson correlation coefficients (r) reflecting the response of plant biomass to cumulative net N mineralization in cucumber rhizosphere soils (*n* = 48).

	Total Microbial Biomass	Bacteria	Fungi	Archaea	*Fusarium oxysporum* f. sp. *cucumerinum*	Net N Mineralization
r	0.515	0.423	0.060	−0.385	−0.390	0.599
*p*-Value	**<0.001 ^a^**	**0.003**	0.685	**0.007**	**0.006**	**<0.001**

^a^ *p*-Values in bold text denote the significant differences among treatments (*p* < 0.05).

## Data Availability

Not applicable.
